# Fibroblasts inhibit osteogenesis by regulating nuclear-cytoplasmic shuttling of YAP in mesenchymal stem cells and secreting DKK1

**DOI:** 10.1186/s40659-023-00481-y

**Published:** 2024-01-20

**Authors:** Fei Huang, Guozhen Wei, Hai Wang, Ying Zhang, Wenbin Lan, Yun Xie, Gui Wu

**Affiliations:** 1 Department of Orthopaedics, The First Affiliated Hospital, Fujian Medical University, No. 20, Chazhong Road, Taijiang District, Fuzhou, 350005 Fujian China; 2 Department of Orthopaedics, Binhai Campus of the First Affiliated Hospital, National Regional Medical Center, Fujian Medical University, Fuzhou, 350212 Fujian China; 3 Central Laboratory, First Affiliated Hospital, Fujian Medical University, Fuzhou, 350005 Fujian China

**Keywords:** Fibroblasts, Osteoblast differentiation, MSCs, Cell competition, YAP

## Abstract

**Background:**

Fibrous scars frequently form at the sites of bone nonunion when attempts to repair bone fractures have failed. However, the detailed mechanism by which fibroblasts, which are the main components of fibrous scars, impede osteogenesis remains largely unknown.

**Results:**

In this study, we found that fibroblasts compete with osteogenesis in both human bone nonunion tissues and BMP2-induced ectopic osteogenesis in a mouse model. Fibroblasts could inhibit the osteoblastic differentiation of mesenchymal stem cells (MSCs) via direct and indirect cell competition. During this process, fibroblasts modulated the nuclear-cytoplasmic shuttling of YAP in MSCs. Knocking down YAP could inhibit osteoblast differentiation of MSCs, while overexpression of nuclear-localized YAP-5SA could reverse the inhibition of osteoblast differentiation of MSCs caused by fibroblasts. Furthermore, fibroblasts secreted DKK1, which further inhibited the formation of calcium nodules during the late stage of osteogenesis but did not affect the early stage of osteogenesis. Thus, fibroblasts could inhibit osteogenesis by regulating YAP localization in MSCs and secreting DKK1.

**Conclusions:**

Our research revealed that fibroblasts could modulate the nuclear-cytoplasmic shuttling of YAP in MSCs, thereby inhibiting their osteoblast differentiation. Fibroblasts could also secrete DKK1, which inhibited calcium nodule formation at the late stage of osteogenesis.

**Supplementary Information:**

The online version contains supplementary material available at 10.1186/s40659-023-00481-y.

## Background

Bone possesses natural self-repair capabilities and can initiate a repair process when fractures occur. During the repair process, a soft callus containing fibrous tissues and cartilaginous tissue initially forms. Later, the soft callus becomes a mature bony callus by intramembranous and endochondral ossification [1]. The newly formed callus can eventually unite the broken bone, leading to fracture healing. However, certain large bone defects or complex fractures may exceed the bones’ inherent repair abilities, resulting in insufficient bony callus generation and bone nonunion. In this context, fibrous tissues overgrow in the gap between the fractured bones, creating a scar-like connection [2]. Nevertheless, scar tissue repair cannot fully restore bone function due to its inability to withstand mechanical stress. Therefore, there are two potential outcomes for the repair of bone fractures: bone repair or fibrous scar repair. However, the interaction of these two repair processes and whether these two types of repair processes compete with one another in vivo remain largely unknown.

Mesenchymal stem cells (MSCs), which can be differentiated into osteoblasts, play important roles in bone repair. Enhancing the homing and osteoblast differentiation of MSCs are major approaches to improve bone regeneration [3–5]. Cytokines such as bone morphogenetic protein 2 (BMP2) have been utilized to promote osteoblast differentiation of MSCs [6]. Fibroblasts are the core components of fibrous scars. Fibroblasts have been reported to inhibit the osteogenic differentiation of MSCs in diverse ways. Kaneda-Ikeda et al. demonstrated that fibroblasts regulate mir-10-3p and ETS variant transcription factor 1 (ETV1) expression to inhibit osteogenic differentiation of MSCs [7]. In addition, dental silver fibroblasts have been shown to directly secrete BMP natural inhibitors, such as Gremlin1, to inhibit preosteoblast osteogenic differentiation [8].

However, few studies have focused on the cell-to-cell interaction between fibroblasts and MSCs in the defective bone environment. Competitive cellular interactions are prevalent in nature. Lower-fitness cells were eliminated, while fitter winner cells survived during competition [9]. However, it remains unclear whether and how fibroblasts compete with MSCs during bone repair to affect the outcome of bone repair.

Our research indicates that fibroblasts inhibit BMP2-mediated osteoblast differentiation of MSCs via cell competition between fibroblasts and MSCs. The effector of the Hippo signalling pathway, Yes-associated protein (YAP)/tafazzin (TAZ), can sense changes in the competition of space and nutrients between cells [10]. Nuclear localized YAP has also been reported to enhance osteoblast differentiation of MSCs [11]. Interestingly, our results show that fibroblasts can regulate the nuclear-cytoplasmic shuttling of YAP in MSCs. Furthermore, constitutively nuclear localized YAP in MSCs can reverse the inhibition of osteogenic differentiation of MSCs caused by fibroblasts. These results indicate that fibroblasts can inhibit MSC osteoblast differentiation by regulating YAP localization in MSCs. In addition, fibroblasts can inhibit osteoblast differentiation of MSCs indirectly via Dickkopf WNT signalling pathway inhibitor 1 (DKK1) secretion. Overall, our research reveals how fibroblasts can regulate osteogenesis directly via cell competition and indirectly via cytokine secretion. YAP and DKK1 can be potential targets for improving bone regeneration in the future.

## Materials and methods

### Reagents

BMP2 (R&D, Minnesota, USA, 355-BM-100), DKK1 (BioLegend, California, USA, 759602), 4′,6-diamidino-2-phenylindole (DAPI, Solarbio, Beijing, PRC, C0060), carboxyfluorescein succinimidyl ester (CFSE, eBioscience, California, USA, 65-0850-84) and gallocyanine (Selleck, Texas, USA, S5602) were used. The antibodies used in this study were monoclonal anti-YAP (Cell Signaling Technology, Boston, USA, 14074), anti-pYAP S127 (Cell Signaling Technology, Boston, USA, 13008), anti-actin alpha2, smooth muscle (anti-α-Sma, Servicebio, Wuhan, PRC, GB111364), anti-osteocalcin (anti-OCN, BioByt, Cambridge, UK, orb259644), tetramethylrhodamine isothiocyanate (TRITC)-conjugated anti-rabbit antibody (ABclonal, Wuhan, PRC, AS040), fluorescein 5-isothiocyanate (FITC)-conjugated anti-mouse antibody (ABclonal, Wuhan, PRC, AS001) and anti-β-actin (Sigma, St. Louis, USA, A1978). Tris-HCl, NaCl and other chemicals were purchased from Sigma.

### Cell culture and differentiation

MSCs were obtained from bone marrow taken from C57BL/6 mice (BMSCs). Total bone marrow cells were cultured in stem cell cultures with 10% foetal bovine serum (FBS) from Cyagen (Guangzhou, PRC). After 48 h, the suspended cells were discarded, and the adherent cells were cultured for 2 weeks to obtain primary mouse BMSCs. The BMSCs used for differentiation assays were at less than eight passages. BMSCs from mice were cultured in differentiation media (α-MEM with 10% FBS, 50 µM ascorbic acid, and 100 mM β-glycerophosphate from Gibco) with 200 ng/mL BMP2 to induce osteoblast differentiation. After BMSCs were cultured in the differentiation media for 7 days, alkaline phosphatase (ALP) staining was conducted using the BCIP/NBT Alkaline Phosphatase Color Development Kit (Beyotime) to examine the early osteoblast differentiation of BMSCs. Furthermore, Alizarin red staining (Solarbio, Beijing, PRC) was conducted to examine the late osteogenic differentiation of BMSCs after BMSCs were cultured in differentiation media for 14–21 days. Mouse embryonic fibroblast (MEF) cells were extracted from 13.5-day embryos of C57BL/6 mice. MEFs, at less than five passages, were cultured in DMEM (Gibco, California, USA) with 10% FBS (Gibco, California, USA).

### CFSE-labelled MSCs

BMSCs were resuspended in 1 mL of PBS with 2.5 µM CFSE. BMSCs were dyed in CFSE solution and shaken in a 37 °C water bath for 10 min. Then, the cells were washed with complete stem culture media twice before the following experiments.

### Adenovirus infection of MSCs

The shRNA for YAP and constitutively active YAP (5SA) DNA were obtained from Cyagen (Guangzhou, PRC) and delivered via adenovirus. The shRNA sequence for YAP was 5′-ATGTGTCTCCAGGAGTAATAA-3′. The mutant form of YAP (5SA) had mutations at S61, S109, S127, S164 and S381. MSCs were infected with either the shRNA for YAP to knock down its expression or the constitutively active YAP (5SA) to overexpress it. Western blot analysis was performed 48–72 h after infection to confirm the expression levels of YAP. Equal numbers of MSCs with YAP knockdown/overexpression or controls were used for further differentiation assays.

### Cell proliferation

MSCs (3 × 10^3^) were cultured in a single well of a 96-well plate for 0 d, 1 d, 2 d and 3 d. Subsequently, cells were harvested and subjected to the CCK-8 assay. Absorbance at 450 nm was examined using a Molecular Devices SpectraMax i3X system. The experiments were conducted three times, and the average relative cell viability to Day 0 was calculated.

Quantitative analysis of cell proliferation under cell coculture conditions was performed as follows. After being labelled with the fluorescent dye CFSE, MSCs were cultured with the indicated treatment. Subsequently, cells were harvested and subjected to flow cytometry using BD Accuri C6. The percentage of MSCs with decreased fluorescence provided an indication of the proportion of proliferating MSCs.

### Cell migration

A total of 5 × 10^3^ MSCs labelled with CFSE and 5 × 10^3^ MEFs in free DMEM were placed on the upper layer of a Corning (New York, USA) cell culture insert with an 8.0 μm polycarbonate membrane. DMEM with 10% FBS and 200 ng/mL BMP2 was placed below the cell permeable membrane. Following an incubation period of 24 h at 37 °C and 5% CO_2_, the cells that had migrated through the membrane were harvested and subjected to immunofluorescence. Images were taken using a Zeiss Axio Scope A1 FL for FISH microscopy.

### Human samples

Human samples were obtained from patients with unhealed fractures over 6 months who were diagnosed with bone nonunion, which required surgical intervention in the clinic. During the operation, tissues in the gap of bone fracture were harvested and stored in formalin solution for further analysis. All experiments were approved by the Ethical Committee of First Affiliated Hospital, Fujian Medical University (MRCTA, ECFAH of FMU [2022]340), and all patients signed informed consent forms. Detailed characteristics of the donors are shown in Additional file 1: Table S1.

### Mice

Female C57BL/6 mice, 6–8 weeks old, used in this study were bred and maintained in a specific pathogen-free animal facility at Fujian Medical University. All animal experiments were approved by the Animal Ethical Committee of Fujian Medical University (FJMU IACUC 2021-0506). To exclude the interference of in situ osteoblasts, we used only an ectopic bone formation model. C57BL/6 mice were anaesthetized with an intravenous injection of 1.5% pentobarbital sodium (40 mg/kg body weight, Sigma, USA). A 1 cm skin incision was made on the skin of each mouse’s back, and the myolemma was longitudinally split. BMP2-loaded collagen materials (7.5 µg BMP2/150 µL 3% rat tail collagen I solution) or control materials (vehicle/150 µL of 3% rat tail collagen I solution) were implanted into the intramuscular gap of the backs of the mice. The control group or the BMP2 group contained five animals each in one experiment. Animals in the same group were fed in the same cage. The operation was performed by the same researcher, and all the mice were maintained in the same environment to minimize potential confounders. The experiment was repeated three times, and a total of 30 mice were used. After the muscles and skin were sutured, 200,000 IU penicillin sodium was injected intramuscularly to prevent possible infection. Animals were sacrificed after 8 weeks, and no animals were excluded from the experiment. Approximately 0.5 cm^3^ tissues around the implanted sites were harvested for further examination.

### Masson staining

The tissues were embedded in paraffin and cut into 2.5 μm tissue sections. Tissue sections were dewaxed with xylene and rehydrated with a gradient of 100%, 95% and 75% alcohol. A Masson staining kit (Solarbio, Beijing, PRC, G1346) was used to stain the sections. Then, a neutral resin was used to mount the sections. Photos were taken using a BX53 Olympus microscope.

### Immunofluorescence

The protocol was the same as that of Masson staining before tissue sections were incubated with the primary antibodies. For cell samples, the samples were harvested and fixed with 4% paraformaldehyde (Sigma, Shanghai, PRC) for 20 min. Then, 10% BSA was used to block the cell samples for 20 min, and 0.25% Triton X-100 was used to permeate the samples for 5 min at room temperature. After that, the samples were incubated with primary antibodies overnight at 4 °C. On the second day, samples were further incubated with TRITC- or FITC-conjugated secondary antibodies for one hour at room temperature. DAPI (Solarbio, Beijing, PRC, C0060) was used to stain the nucleus. Images were taken using a Zeiss LSM 800 laser scanning confocal microscope.

### Immunoblotting

Cells were lysed with TNE buffer (10 mM Tris-HCl, 150 mM NaCl, 1 mM EDTA, 0.5% NP40, pH = 7.5) for the immunoblotting assay. The cell lysates were mixed with 4× loading buffer (40 mM Tris-HCl, 200 mM DTT, 4% SDS, 40% glycerol, 0.032% bromophenol blue, pH = 8.0) and run with 4% stacking gel and 10% separating gels. Proteins on the gels were transferred to nitrocellulose filter membranes for antibody incubation. The membrane exposure was performed using the Thermo Pierce ECL and FluorChem E (Protein Simple, California, USA) System.

### Quantitative real-time PCR

Total cell RNA was isolated using TRIzol (Invitrogen, California, USA), and cDNA was synthesized using Revertra Ace (Promega, Madison, USA). Real-time PCR was performed using an ABI QuantStudio 5 system. The expression level of genes was measured using the comparative Ct method. Expression values were normalized to glyceraldehyde-3-phosphate dehydrogenase (*Gapdh*) expression. The primer sequences are shown Additional file 1: Table S2.

### RNA-seq

The RNA-seq data were obtained from the Illumina HiSeq platform (Illumina, Inc., San Diego, CA, USA). RNA library preparation was performed as described in the instructions provided in the QIAseq Stranded RNA Library Kits (QIAGEN, Dusseldorf, Germany). Total RNA was extracted from cells, and the mRNA was fragmented to an average insert size of 200–400 bp. The resulting RNA fragments were then converted into first-strand cDNA using reverse transcriptase (Thermo Fisher Scientific, Massachusetts, USA) and random primers. The first-strand cDNA was further converted into double-stranded DNA in the presence of dUTP, and these cDNA fragments were subjected to the addition of a single ‘A’ base and subsequent ligation of the adapter. The products were purified and enriched via PCR to generate the final library. The libraries were sequenced on the Illumina HiSeq platform (Illumina, Inc., San Diego, CA, USA). Raw sequences were mapped to the mouse genome mm10 by STAR (v2.5.4b_ENREF_32) [12]. The expression level FPKM values were obtained from Cuffnorm in the Cufflinks package (v2.2.1) [13]. The data could be assessed online at the Gene Expression Omnibus (GEO) (No. GSE205156).

### ELISA

For ELISAs, 1 × 10^4^ MSCs and MEFs were cultured in 1 mL of medium with 2% FBS. Supernatants from cell cultures were collected as samples and subjected to DKK1 ELISAs (Multi Sciences, Hangzhou, PRC, EK2111).

### Statistical analysis

Statistical analysis was performed using Student’s t test, one-way ANOVA and two-way ANOVA. *P* values < 0.05 were considered statistically significant.

## Results

### The formation of fibrous scars could compete with BMP2-mediated osteogenesis

**Fig. 1:**
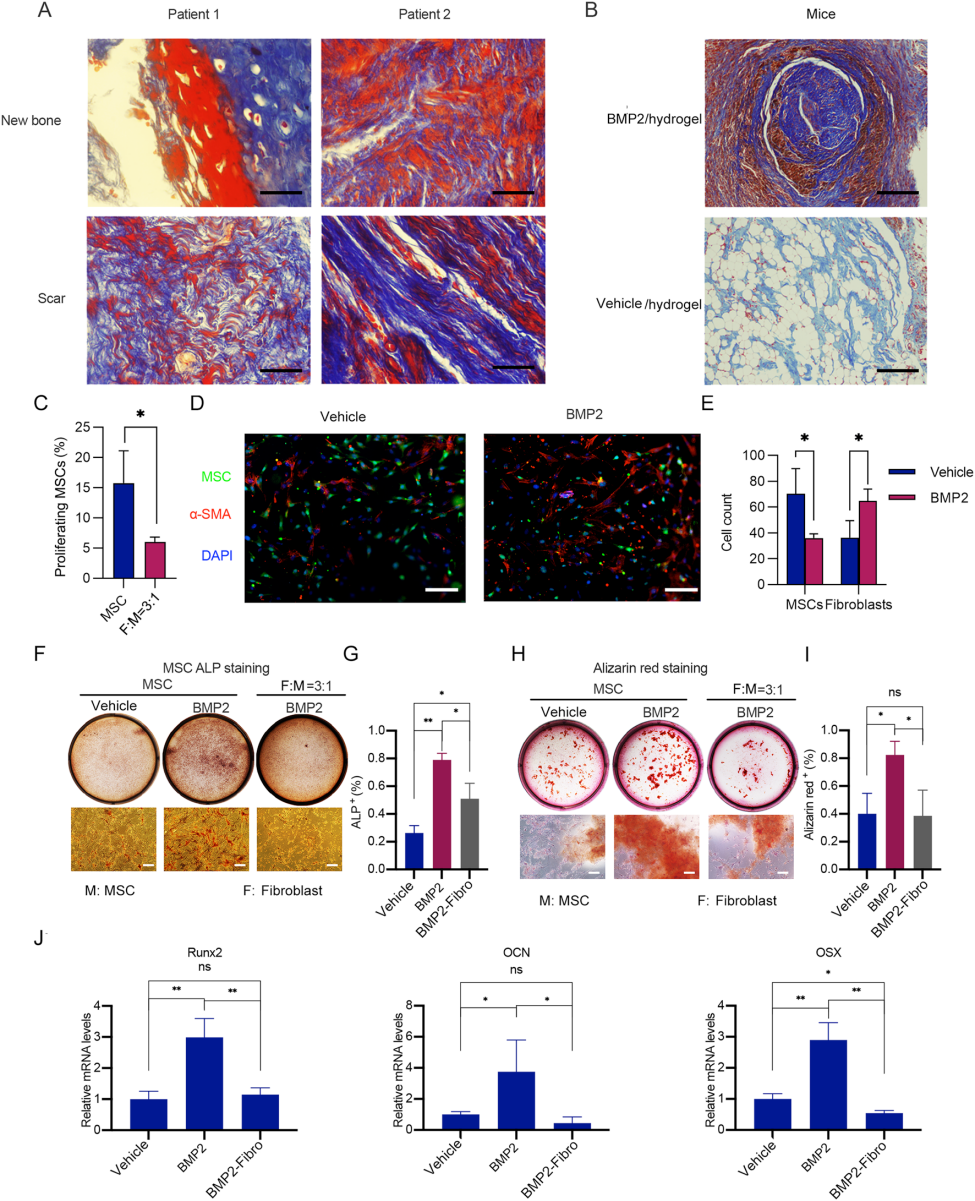
Assessment of the roles that fibrous tissues and fibroblasts played on osteogenesis. **A** Masson staining was performed on tissues harvested from patients with bone nonunion. Blue staining indicated the presence of fibrous tissues while the red staining indicated the presence of newly formed bone. Scale bars of the ×100 photos were 100 μm. **B** Tissues obtained from the experiments of BMP2 induced ectopic bone formation were subjected to Masson staining. Blue staining represented fibrous tissues and the red staining indicated the presence of newly formed bone. Scale bars of the ×100 photos were 100 μm. **C** After being labelled with 2.5 µM CFSE (green fluorescence), a total of 5 × 10^3^ MSCs were cultured either alone or with 1.5 × 10^4^ fibroblasts in a single well of a 24-well plate for 72 h. Subsequently, the cells were harvested and subjected to flow cytometry. Cells exhibiting green fluorescence signals lower than parental cells were indicative of proliferating cells. Three independent experiments were conducted, and the average percentages of proliferating MSCs were calculated and shown (*P < 0.05). *M* MSC, *F* fibroblast. **D** After being labelled with 2.5 µM CFSE (green fluorescence), a total of 5 × 10^3^ MSCs were cultured with an equal number of fibroblasts in free DMEM on the upper layer of Corning cell culture insert with 8.0 μm polycarbonate membrane. The complete culture media, supplemented either with a vehicle or with 200 ng/mL BMP2, was placed below the cell permeable membrane. Representative immunofluorescent staining images of the fibroblasts and MSCs that migrated through the Transwell membrane were presented. Green cells indicated MSCs, red cells indicated α-SMA^+^ cells (fibroblasts), and blue colour indicated DAPI, a marker of nucleus. Scale bars, 100 μm. **E** Quantitative analysis of the numbers of MSCs and fibroblasts in **C** was conducted. Cell numbers of at least three fields were counted and average cell numbers were calculated (*P < 0.05; **P < 0.01). **F** A total of 1 × 10^4^ MSCs were cultured either alone or with 3 × 10^4^ fibroblasts in a single well of a 12-well plate. The cells were treated either with a vehicle or with 200 ng/mL BMP2 for 7 days. Subsequently, the cells were harvested and subjected to ALP staining. Representative images of the wells and cells were presented. Scale bars, 100 μm. *M* MSC, *F* fibroblast.** G** The percentages of ALP^+^ cells in **E** were quantitatively analysed. The average percentages of ALP^+^ cells were calculated from at least three fields and shown (*P < 0.05; **P < 0.01). **H** 1 × 10^4^ MSCs were cultured either alone or with 3 × 10^4^ fibroblasts in a single well of a 12-well plate. The cells were treated either with a vehicle or with 200 ng/mL BMP2 for 14 days. Subsequently, the cells were harvested and subjected to Alizarin red staining. Representative images of the wells and cells were presented. Scale bars, 100 μm. **I** The Alizarin red^+^ area percentages in **G** were quantitatively analysed. The average percentages of Alizarin red^+^ areas were calculated from at least three fields and presented (*P < 0.05; **P < 0.01). *M* MSC, *F* fibroblast. **J** MSCs and fibroblasts were either cultured alone or cocultured as indicated in **I**. Subsequently, the cells were harvested and the total RNAs were extracted. cDNAs were synthesized and subjected to qPCR. Relative mRNA levels of *Runx2*, *Ocn* and *Osx* to *Gapdh* in MSCs were calculated and presented (*P <  0.05; **P < 0.01)

Bone defects can be repaired through complete bone regeneration or by connecting remaining bone fragments using soft tissues. Specifically, we examined the soft tissues harvested from patients who had experienced bone nonunion after fractures occurred for over 6 months. Masson staining showed that most of the newly regenerated bone tissues (stained red) were located at the boundary of bone nonunion. However, fibrous scars (stained blue) comprised the main components of the soft tissues derived from bone nonunion, where the regeneration of bones was not active (Fig. 1A). These results indicated that competition may exist between bone regeneration and the formation of fibrous scars during the repair of bone fractures. We further analysed the mechanisms involved in this competition in a mouse model and in vitro cell culture models.

Diverse types of growth factors contribute to bone regeneration when fractures occur. BMP2 is the most important osteogenic factor for inducing osteoblast differentiation in MSCs and preosteoblasts [14, 15] and has been utilized to promote bone regeneration in clinical settings. To investigate the potential competition between bone regeneration and the formation of fibrous scars during BMP2-induced osteogenesis, we utilized a mouse model of BMP2-induced ectopic osteogenesis. This approach allowed us to eliminate the influence of other growth factors that had effects on bone fracture healing from the typical microenvironment. BMP2-carried collagen materials were implanted into the intramuscular gap of the mouse back, and ectopic osteogenic tissues were formed after 8 weeks. Masson staining revealed that both new bone tissues (stained red) and fibrous tissues (stained blue) formed in BMP2-induced ectopic osteogenic tissues (Fig. 1B). Consistent with the results of patient samples, the newly regenerated bone tissue was at the boundary of the ectopic osteogenic tissue, while the dense fibrous tissue was in the core area where osteogenesis was not active.

First, we analysed the potential competition between MSCs and fibroblasts in cell proliferation. To achieve this, we labelled MSCs with the fluorescent dye CFSE and cultured them directly with fibroblasts. As cell proliferation occurred, the total fluorescence intensity within MSCs decreased due to CFSE derived from the parental cells being equally distributed among daughter cells. The percentage of MSCs that demonstrated decreased fluorescence provided an indication of the proportion of proliferated MSCs. As demonstrated in Fig. 1C, fibroblasts were found to inhibit the proliferation of MSCs, suggesting a competitive relationship between the two cell types. The migration and differentiation of MSCs at the fracture site promotes the healing of bone defects [3]. Thus, we further analysed the competition between fibroblasts and MSCs in cell migration and differentiation. We cocultured fibroblasts and MSCs on the upper layer of the cell culture insert of the Transwell under BMP2 treatment, where MSCs were labelled with CFSE (green) and fibroblasts were labelled with α-Sma (red). As shown in Fig. 1D, E, more fibroblasts migrated through the membrane following treatment with BMP2 than the control, while the number of migrated MSCs decreased. These results suggested that BMP2 promoted the migration of fibroblasts while having a comparatively inhibitory effect on the migration of MSCs when these two types of cells were cocultured. Fibroblasts might compete with MSCs in BMP2-mediated cell migration.

To further determine the effect of fibroblasts on BMP2-mediated osteoblast differentiation of MSCs, we performed direct coculture of fibroblasts and MSCs. ALP staining showed that the coculture of fibroblasts and MSCs could inhibit BMP2-induced early osteogenic differentiation of MSCs (Fig. 1F, G). Consistently, calcium nodule formation during late-stage osteoblast differentiation of MSCs, represented by Alizarin red staining, could also be suppressed by fibroblasts (Fig. 1H, I). Additionally, the expression of the osteogenesis-related genes *Runx2*, *Ocn* and *Osx* at Day 14 showed that BMP2 could induce the osteoblast differentiation of MSCs and that the coculture of MSCs and fibroblasts could inhibit differentiation (Fig. 1J). Taken together, the above results revealed that fibrous tissue may competitively inhibit osteogenesis during bone regeneration. Fibroblasts, as the main effector cells of fibrous scars, could inhibit BMP2-induced osteogenic differentiation of MSCs.

### Fibroblasts could inhibit osteoblast differentiation of MSCs in vitro

**Fig. 2:**
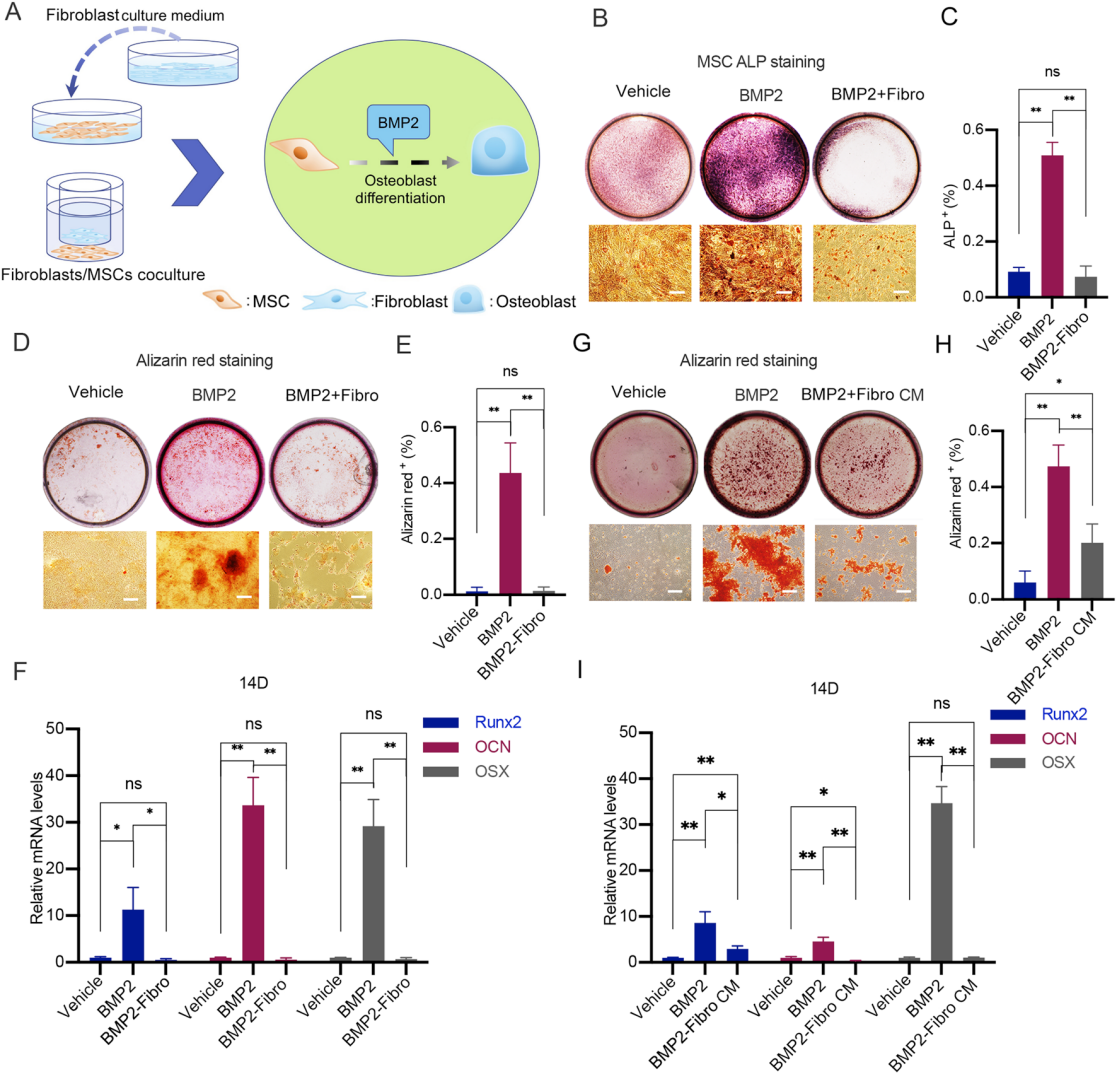
The effects of fibroblasts and conditional media from fibroblasts on BMP2 induced osteoblast differentiation of MSCs. **A** Schematic illustration showed the experimental protocols. MSCs were directly co-cultured with conditional media from fibroblasts or indirect co-cultured with fibroblasts in Transwell. BMP2 was utilized to induce osteoblast differentiation of MSCs. **B** A total of 3 × 10^4^ fibroblasts were cultured on the upper layer of the Corning cell culture insert with 0.4 μm polyester membrane. 1 × 10^4^ MSCs were placed below the cell permeable membrane for 7 days, supplemented either with a vehicle or with 200 ng/mL BMP2. Subsequently, the cells were harvested and subjected to ALP staining. Representative images of the wells and cells were presented. Scale bars, 100 μm. **C** The percentages of ALP^+^ cells in **B** were quantitatively analysed. The average percentages of ALP^+^ cells were calculated from at least three fields and shown (**P < 0.01). **D** A total of 3 × 10^4^ fibroblasts were cultured on the upper layer of Corning cell culture insert with 0.4 μm polyester membrane. 1 × 10^4^ MSCs were placed below the cell permeable membrane for 14 days, supplemented either with a vehicle or with 200 ng/mL BMP2. Subsequently, the cells were harvested and subjected to Alizarin red staining. Representative images of wells and cells were presented. Scale bars, 100 μm. **E** The percentages of Alizarin red^+^ areas in **D** were quantitatively analysed. The average percentages of Alizarin red^+^ areas were calculated from at least three fields and presented (**P < 0.01). **F** MSCs were treated as indicated in **D**. Subsequently, the cells were harvested and total RNAs were extracted. cDNAs were synthesized and subjected to qPCR. Relative mRNA levels of *Runx2*, *Ocn* and *Osx* to *Gapdh* in MSCs were calculated and presented (*P < 0.05; **P < 0.01). **G** 1 × 10^4^ MSCs were cultured with conditional media from fibroblasts and treated either with 200 ng/mL BMP2 or the control vehicle for 14 days. Subsequently, the cells were harvested and subjected to Alizarin red staining. Representative images of wells and cells were presented. Scale bars, 100 μm. **H** The percentages of Alizarin red^+^ areas in **G** were quantitatively analysed. The average percentages of Alizarin red^+^ areas were calculated from at least three fields and presented (**P < 0.01). **I** MSCs were treated as indicated in **G**. Subsequently, cells were harvested and the total RNAs were extracted. cDNAs were synthesized and subjected to qPCR. Relative mRNA levels of *Runx2*, *Ocn* and *Osx* to *Gapdh* in MSCs were calculated and presented (*P < 0.05; **P < 0.01)

Fibroblasts could inhibit osteoblast differentiation of MSCs through direct contact based on the results above. We then investigated whether the inhibitory effects were mediated through direct cell‒cell contact or diffusible cytokines secreted by cells. To answer this question, we conducted a Transwell assay to spatially separate fibroblasts and MSCs while allowing the free exchange of cytokines and nutrients in the culture media (Fig. 2A). In this Transwell coculture condition, the presence of fibroblasts could still inhibit BMP2-mediated osteoblast differentiation of MSCs (Fig. 2B, F), indicating that fibroblasts inhibited the osteoblast differentiation of MSCs independent of direct cell‒cell contacts.

We further examined whether fibroblasts inhibited osteoblast differentiation of MSCs via secretion of cytokines. Interestingly, it was found that conditioned media from fibroblasts containing cytokines secreted by fibroblasts could only inhibit the late osteogenic differentiation of MSCs but had no effect on early differentiation or cell proliferation (Fig. 2G–I and Additional file 1: Fig. S1A–C). Taken together, our results suggested that the impact of fibroblasts on BMP2-mediated osteoblast differentiation of MSCs did not rely on direct cell‒cell contact. Cytokines secreted by fibroblasts could affect osteoblast differentiation of MSCs at a late stage while having minimal effects on the early differentiation of MSCs. Therefore, fibroblasts could inhibit osteoblast differentiation of MSCs through both direct and indirect contact in vitro.

### Fibroblasts could induce nuclear-cytoplasmic shuttling of YAP in MSCs via cell competition

**Fig. 3:**
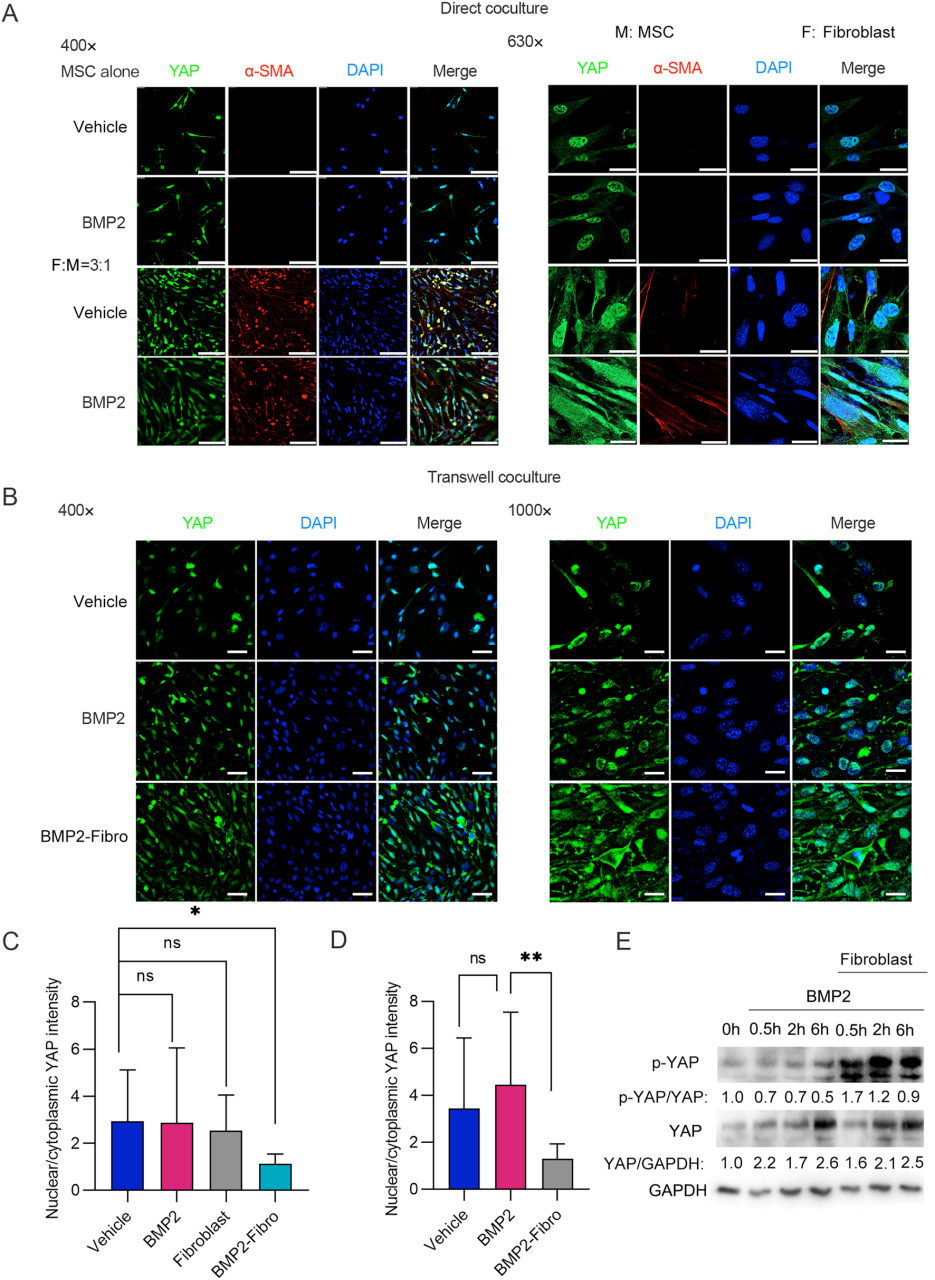
The nuclear-cytoplasmic shuttling of YAP in MSCs after co-cultured with fibroblasts. **A** A total of 1 × 10^4^ MSCs were cultured either alone or with 3 × 10^4^ fibroblasts in a single well of a 12-well plate. The cells were treated either with a vehicle or with 200 ng/mL BMP2 for 2 h. Subsequently, the cells were harvested and subjected to immunofluorescence. Representative immunofluorescent staining images were shown. The colour green was used to represent YAP, while red indicated α-SMA and blue represented DAPI. Scale bars, 100 μm for ×400, 20 μm for ×630. *M* MSC, *F* fibroblast. **B** 3 × 10^4^ fibroblasts were cultured on the upper layer of Corning cell culture insert with 0.4 μm polyester membrane. 1 × 10^4^ MSCs were placed below the cell permeable membrane for 2 h, supplemented either with a vehicle or with 200 ng/mL BMP2. Subsequently, the cells were harvested and subjected to immunofluorescence. Representative immunofluorescent staining images of MSCs were presented. Green colour represented YAP, while blue indicated DAPI. Scale bars, 25 μm for ×400, 10 μm for ×630. **C**, **D** Quantitative analysis of nuclear/cytoplasmic YAP intensity of MSCs in **A** and **B** was shown. The average nuclear/cytoplasmic YAP intensity of at least ten cells were calculated and presented (*P < 0.05; **P < 0.01). **E** 3 × 10^4^ fibroblasts were cultured on the upper layer of Corning cell culture insert with 0.4 μm polyester membrane. 1 × 10^4^ MSCs were placed below the cell permeable membrane for the indicated time point, supplemented either with a vehicle or with 200 ng/mL BMP2. Subsequently, the cells were harvested and subjected to immunoblotting. Images of western blots to show the expression of YAP and p-YAP in MSCs were presented. Relative band intensity was shown below the gels and full-length gels were presented in Additional file 1: Fig. S7A

The Hippo signalling pathway effector YAP/TAZ is capable of sensing changes in nutrient and space competition between cells [16, 17]. When Hippo signalling is inactivated, YAP/TAZ can regulate the transcription of downstream target genes with TEAD in the nucleus [18]. However, during tissue overgrowth, Hippo signalling is activated, and the kinase LATS1/2 can phosphorylate YAP/TAZ, leading to the cytoplasmic translocation of YAP/TAZ. This nuclear-cytoplasmic shuttling of YAP results in the inhibition of YAP/TAZ activity, which further attenuates cell proliferation [18]. Recent studies have found that the activation of Hippo signalling also regulates cell competition during stem cell differentiation and tissue regeneration [19–21]. Thus, we analysed whether fibroblasts inhibited the osteogenic differentiation of MSCs via YAP. Our direct coculture experiments demonstrated that fibroblasts could enhance the cytoplasmic translocation of YAP in MSCs (Fig. 3A, C). In addition, our Transwell experiments showed that fibroblasts could induce cytoplasmic translocation of YAP from the nucleus in MSCs without direct cell contact (Fig. 3B, D). Furthermore, we observed that BMP2 treatment induced dephosphorylation of YAP S127 (the target of LATS1/2) half an hour after coculture and lasted for six hours in MSCs. However, fibroblasts could increase the pYAP/YAP ratio and contribute to the phosphorylation process, leading to the cytoplasmic translocation of YAP in MSCs (Fig. 3E). This result suggested that BMP2 signalling might help sustain the nuclear localization of YAP in MSCs, but fibroblasts could reverse this process and induce YAP cytoplasmic translocation. Thus, fibroblasts could induce nuclear-cytoplasmic shuttling of YAP in MSCs.

### Fibroblasts inhibited osteoblast differentiation by enhancing the cytoplasmic localization of YAP in MSCs

**Fig. 4:**
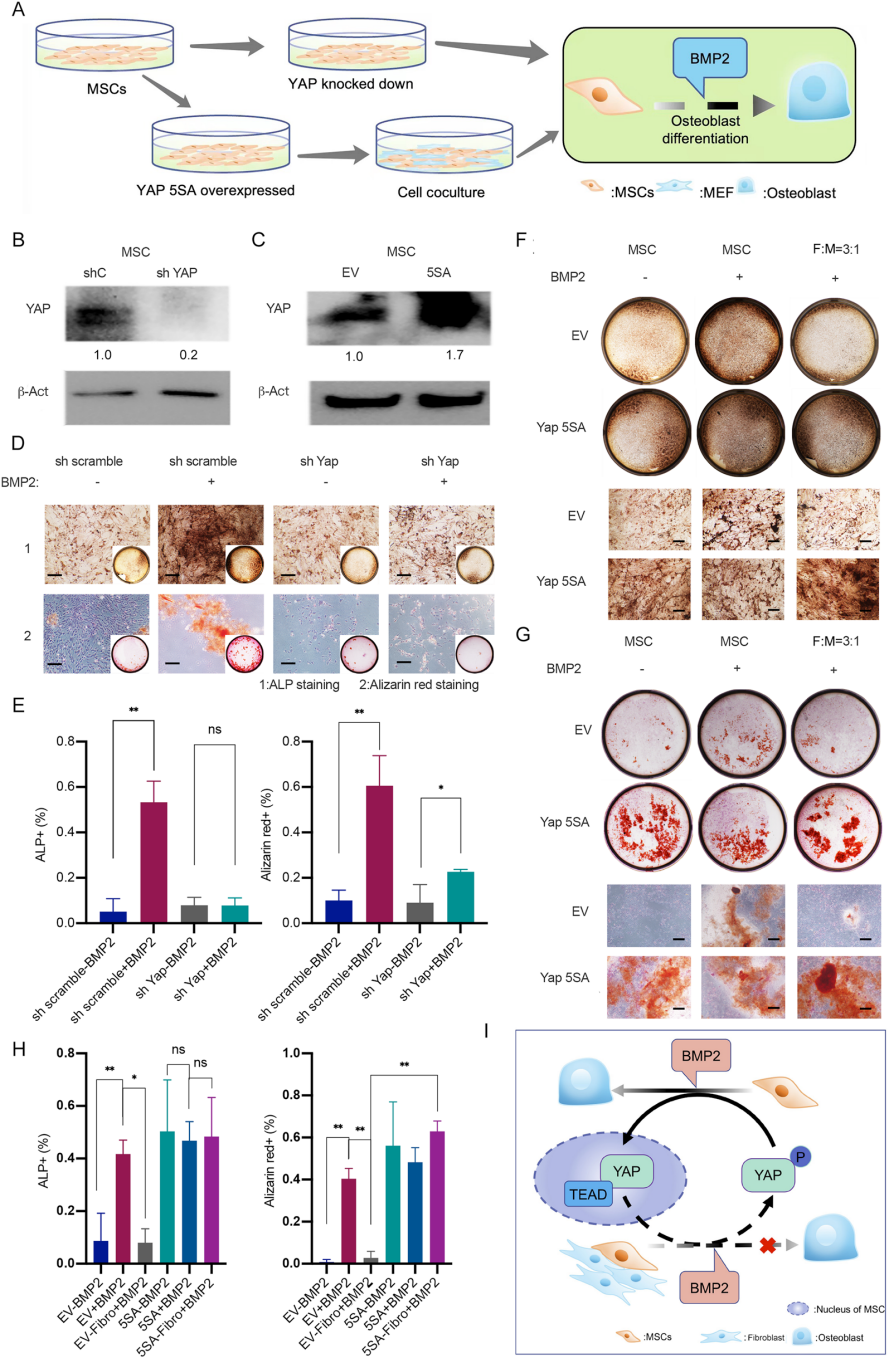
The effects of overexpression and knockdown of YAP on BMP2 induced osteoblast differentiation of MSCs. **A** Schematic illustration showed the experimental protocols. After knocking down YAP, BMP2 was utilized to induce osteogenic differentiation in MSCs for the purpose of studying the effects of YAP on osteoblast differentiation. Moreover, the study also evaluated whether overexpression of the constitutively activated YAP could reverse the inhibitory effect of fibroblasts on MSC osteogenic differentiation. **B**, **C** YAP and β-Actin were detected through western blot following the transfection of MSCs with adenovirus encoding shRNA of YAP (**B**) or YAP-5SA (**C**). The relative band intensity was shown below the gels and full-length gels were presented in Additional file 1: Fig. S7B, C. **D** After MSCs were transfected with either shRNA of YAP or scramble, 1 × 10^4^ MSCs were cultured in a single well of a 12-well plate. After the cells were treated either with a vehicle or with 200 ng/mL BMP2 for 7 days, cells were harvested and subjected to ALP staining. After the cells were treated either with a vehicle or with 200 ng/mL BMP2 for 14 days, cells were harvested and subjected to Alizarin red staining. Representative images of wells and cells were presented. Scale bars, 100 μm. **E** Quantitative analysis of the percentage of ALP^+^ MSCs and Alizarin red^+^ areas of **D** was conducted. The average percentages of ALP^+^ MSCs and Alizarin red^+^ areas were calculated from at least three fields and presented (*P<0.05; **P < 0.01). **F** After being transfected with adenovirus encoding YAP-5SA, MSCs were co-cultured with fibroblasts under either the BMP2 treatment or the vehicle treatment for 7 days. Subsequently, the cells were harvested and subjected to ALP staining. The representative images of the wells and cells were presented. Scale bars, 100 μm. *M* MSC, *F* fibroblast. **G** After being transfected with adenovirus encoding YAP-5SA, MSCs were co-cultured with fibroblasts under either the BMP2 treatment or the vehicle treatment for 14 days. Subsequently, cells were harvested and subjected to Alizarin red staining. The representative images of wells and cells were presented. Scale bars, 100 μm. *M* MSC, *F* fibroblast. **H** Quantitative analysis of the percentages of ALP^+^ MSCs and Alizarin red^+^ areas of **F** and **G** was conducted. The average percentages of ALP^+^ MSCs and Alizarin red^+^ areas were calculated from at least three fields and presented (*P < 0.05; **P < 0.01). **I** Schematic illustration showed how fibroblasts regulated the osteoblast differentiation of MSCs via modulating the nuclear-cytoplasmic shuttling of YAP. Nucleus localized YAP could favour osteoblast differentiation of MSCs under the BMP2 treatment. Fibroblasts could induce the cytoplasmic localization of YAP in MSCs to inhibit osteoblast differentiation of MSCs

YAP/TAZ has been reported to play important roles in osteogenesis [18, 22, 23]. Therefore, we further analysed whether fibroblasts inhibited osteoblast differentiation of MSCs through the modulation of YAP localization in MSCs (Fig. 4A). We knocked down YAP expression and overexpressed nuclear localized YAP (YAP-5SA), which could not be phosphorylated by LATS1/2 [24], in MSCs (Fig. 4B, C). The results showed that knockdown of YAP expression inhibited cell proliferation and BMP2-induced osteogenic differentiation of MSCs (Fig. 4D, E, Additional file 1: Fig. S2A–C). Additionally, overexpression of nuclear-localized YAP (YAP-5SA) enhanced the osteoblast differentiation of MSCs even without BMP2 treatment (Fig. 4F–H), indicating the importance of YAP in the osteoblast differentiation of MSCs. Importantly, our findings revealed that overexpression of the nuclear localized YAP (YAP-5SA) in MSCs could reverse the fibroblast-mediated inhibition of the osteogenic differentiation of MSCs (Fig. 4F–H), supporting the hypothesis that fibroblasts inhibited osteoblast differentiation of MSCs through modulation of nuclear-cytoplasmic shuttling of YAP in MSCs (Fig. 4I).

### Fibroblasts inhibited osteoblast differentiation of MSCs indirectly through the secretion of DKK1

**Fig. 5:**
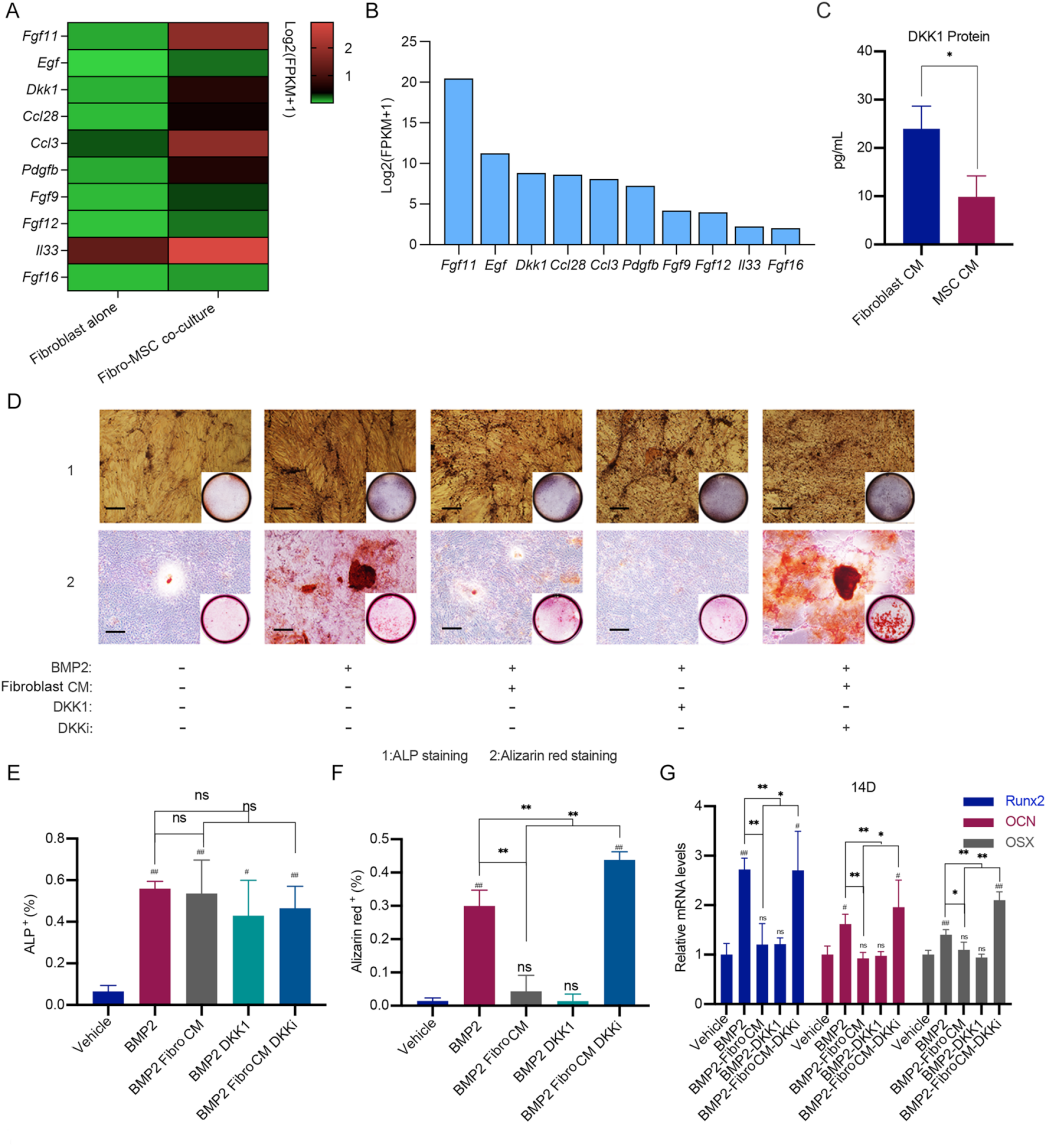
The effects of DKK1 secreted by fibroblasts on the osteoblast differentiation of MSCs. **A** Heatmap with mRNA-based expression of representative genes (cytokines) in fibroblasts either cultured alone or co-cultured with MSCs. The gene names were shown on the right of the heatmap. **B** Histogram showed mRNA-based expression of representative genes (cytokines) in fibroblasts either cultured alone or co-cultured with MSCs. **C** Conditional media obtained from either 1 × 10^4^ fibroblasts or MSCs were collected and analysed with Elisa assay to determine the concentrations of DKK1 (*P < 0.05). **D** After 1 × 10^4^ MSCs were treated with conditional media from fibroblasts, 10 ng/mL DKK1 or 5 µM gallocyanine (DKKi) for 7 days, cells were harvested and subjected to ALP staining. After the cells were treated with conditional media from fibroblasts, 10 ng/mL DKK1 or 5 µM gallocyanine for 14 days, cells were harvested and subjected to Alizarin red staining. 200 ng/mL BMP2 was utilized to induce osteoblast differentiation of MSCs. The representative images of the wells and cells were presented. Scale bars, 100 μm. **E**, **F** The percentages of ALP^+^ cells (**E**) and Alizarin red^+^ areas (**F**) in **D** were quantitatively analysed. The average percentages of ALP^+^ MSCs and Alizarin red^+^ areas were calculated from at least three fields and presented (^#^represented comparison between indicated group and negative control; ^#^*P < 0.05*; ^##^*P < 0.01*; **P < 0.01). **G** MSCs were treated as indicated in **D**. Subsequently, the cells were harvested and total RNAs were extracted. cDNAs were synthesized and subjected to qPCR. Relative mRNA levels of *Runx2*, *Ocn* and *Osx* to *Gapdh* in MSCs were calculated and presented (^#^represented comparison between indicated group and negative control; ^#^*P < 0.05*; ^##^*P < 0.01*; *P < 0.05; **P < 0.01)

The results above showed that fibroblasts inhibited osteoblast differentiation of MSCs at the late stage via cytokines secreted by fibroblasts. We found that conditioned medium from fibroblasts did not affect the nuclear localization of YAP (Additional file 1: Fig. S3A, B), suggesting that cytokines from fibroblasts did not play roles in osteoblast differentiation of MSCs via modulation of YAP localization. Thus, we further performed RNA-Seq to analyse which type of cytokines secreted by fibroblasts had direct functions in MSC differentiation. RNA-seq was carried out to analyse the transcriptome differences among fibroblasts cultured alone and fibroblasts cocultured with MSCs. Our results revealed that DKK1 was one of the cytokines upregulated when fibroblasts were cocultured with MSCs compared with fibroblasts cultured alone (Fig. 5A, B). Furthermore, fibroblasts secreted more DKK1 than MSCs when the cell number was the same (Fig. 5C). DKK1 is a natural inhibitor of canonical Wnt/β-catenin signalling [25, 26], which plays important roles in osteogenesis and bone regeneration [27, 28]. We chose to investigate DKK1 further among the cytokines found because of its relevance to osteogenesis. Consistently, DKK1 and its inhibitor had no effect on the early osteoblast differentiation of MSCs but inhibited the late osteogenic differentiation of MSCs. However, treatment with the DKK1 inhibitor gallocyanine reversed the inhibition caused by conditioned medium from fibroblasts on the late osteogenic differentiation of MSCs (Fig. 5D–G). These results indicated that fibroblasts secreted DKK1 to inhibit the osteogenic differentiation of MSCs at the late stage. In addition, DKK1 and its inhibitor gallocyanine did not affect localization of YAP (Additional file 1: Fig. S3C, D). This result demonstrated that DKK1 inhibited osteogenesis independently of the functions of YAP.

### The accumulation of fibrous tissues was correlated with YAP nuclear-cytoplasmic shuttling in preosteoblasts and reduced osteogenesis in vivo

**Fig. 6:**
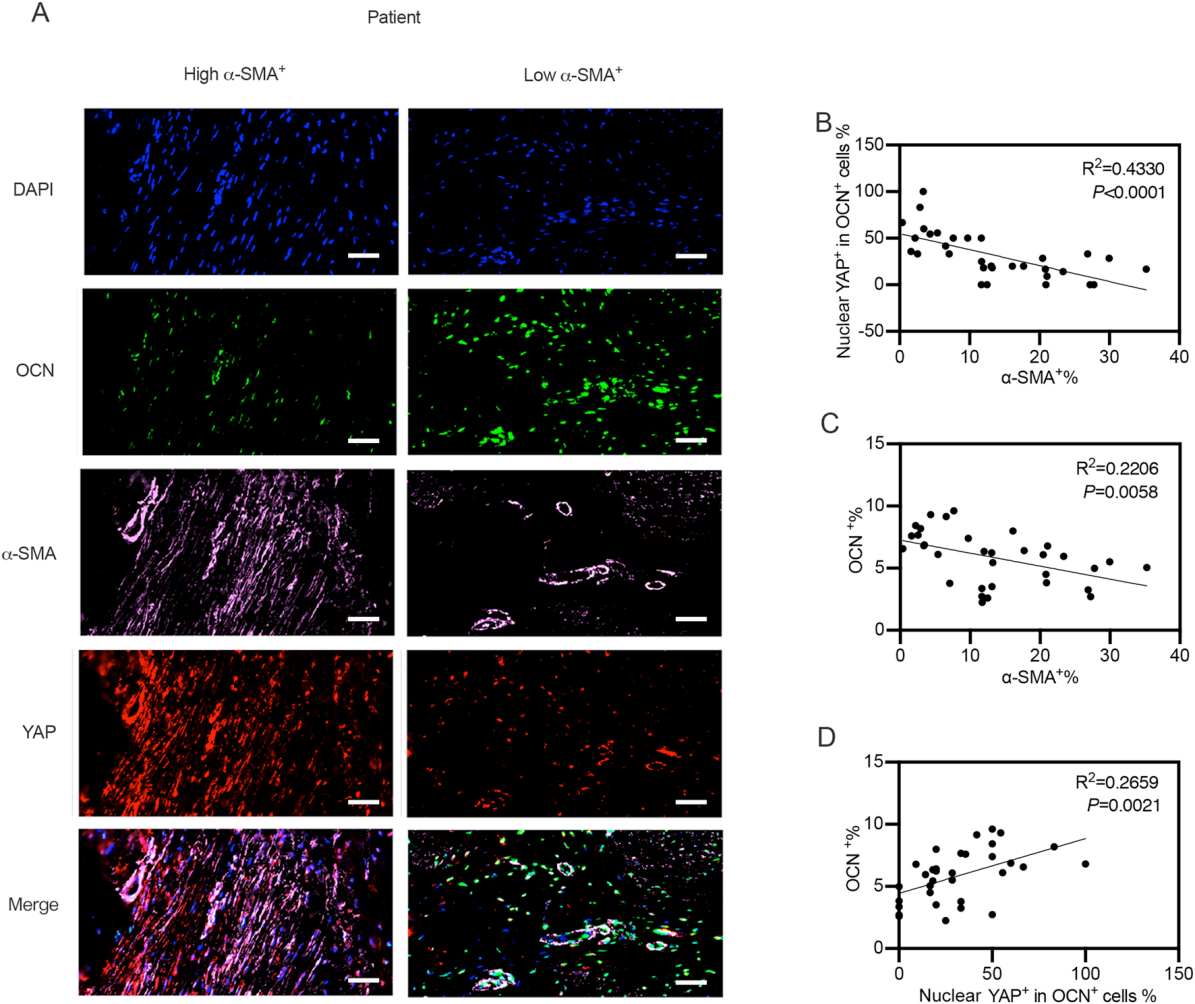
The correlations among fibrous tissues accumulation, osteogenesis and YAP nuclear-cytoplasmic shuttling in vivo. **A** Tissues obtained from patients with nonunion of fractures were harvested and sliced into multiple sections. These sections were subsequently divided into several small unites to enable thorough analysis following immunofluorescent staining. If the α-SMA positive area was over 50%, the area was classified as the high α-SMA expression area, while less than 50% was considered as low expression. Representative immunofluorescent staining images either with high or with low α-SMA^+^ expression were shown. DAPI, OCN and YAP were also stained as indicated in the images. Scale bars, 100 μm. **B** The nuclear and cytoplasmic YAP intensity in OCN positive cells was calculated using the software Image J. Nuclear YAP positive cells were defined as cells in which the nuclear YAP intensity/cytoplasmic YAP intensity was over 1. Scatter diagram illustrated the correlation between the percentage of α-SMA^+^ areas and the percentage of nuclear YAP^+^ in OCN^+^ cells in **A**. We ensured accuracy by having at least three different experimenters analyse all images, with the average value of each experiment being shown. **C** Scatter diagram illustrated the correlation between the percentage of α-SMA^+^ areas and the percentage of OCN^+^ areas in **A**. We ensured accuracy by having at least three different experimenters analyse all images, with the average value of each experiment being shown. **D** The nuclear and cytoplasmic YAP intensity in OCN positive cells were calculated using the software Image J. Nuclear YAP positive cells were defined as cells in which the nuclear YAP intensity/cytoplasmic YAP intensity was over 1. Scatter diagram illustrated the correlation between the percentage of OCN^+^ areas and the percentage of nuclear YAP^+^ in OCN^+^ cells in **A**. We ensured accuracy by having at least three different experimenters analyse all images, with the average value of each experiment being shown

The results above showed that fibroblasts could inhibit osteogenic differentiation of MSCs through modulation of YAP nuclear-cytoplasmic shuttling in MSCs in vitro. To investigate whether fibrous tissues inhibited osteogenesis by modulating nuclear-cytoplasmic shuttling in preosteoblasts in vivo, we used α-Sma to label fibrous tissues and OCN to label preosteoblasts in tissues derived from patients with bone nonunion. If the α-SMA-positive area was over 50%, the area was classified as a high α-SMA expression area, while less than 50% was considered low expression (Fig. 6A and Additional file 1: Fig. S4). The nuclear and cytoplasmic YAP intensities in OCN-positive cells were also calculated. Nuclear YAP-positive cells were defined as cells in which the nuclear YAP intensity/cytoplasmic YAP intensity was over one. We found that the percentages of OCN-positive cells with YAP in the nucleus were negatively correlated with the expression levels of α-Sma (Fig. 6A, B). Additionally, the expression levels of OCN were negatively correlated with the expression levels of α-Sma (Fig. 6A, C). Most OCN-positive cells had YAP located in the nucleus (Fig. 6D). The high α-Sma expressing areas were typically where fibrous tissues were the main components, whereas low α-Sma expressing areas were usually where osteogenesis was active. Consistently, the same phenomena could be observed in BMP2-induced ectopic osteogenic tissues from mice (Additional file 1: Figs. S5 and S6). These findings indicated that the accumulation of fibrous tissues was correlated with YAP nuclear-cytoplasmic shuttling in preosteoblasts and reduced osteogenesis in vivo.

## Discussion

The development of fibrosis is a common outcome of tissue repair responses after injuries. When injuries occur, local tissue fibroblasts become activated. Fibroblasts usually play essential roles in the secretion of cytokines and synthesis of ECM components, which initiates wound healing and tissue regeneration [29]. When damage is minor, the repair response is efficient, resulting in the quick elimination of excess ECM components and the restoration of normal tissue architecture. However, when injury is severe, the normal tissue architecture cannot be reconstructed efficiently, and fibrous scars form, which can lead to organ dysfunction and ultimately organ failure [30, 31]. Fibrosis has been known for years to cause major harm to organs, such as the lung, liver, kidney, heart, and skin [30, 32–35]. However, few studies have focused on the consequences of fibrous scarring on bone dysfunction at sites of large and complex bone defects. In this research, we investigated the detailed mechanism by which fibroblasts inhibited osteogenesis during the repair of severe bone defects and defined ‘fibrosis’ as a process that could occur in bone nonunion and harm osteogenesis.

According to the results of our research, fibroblasts inhibited osteogenesis through cell competition with MSCs. Cell competition plays important roles in development, tissue haemostasis and tumorigenesis [20, 36, 37]. Cell competition is frequently a short-range intercellular communication between neighbouring cells, in which cells compare their fitness with that of their neighbours and eliminate the cells with relatively lower fitness, resulting in a ‘winner type’ and a ‘loser type’ [9]. The Hippo signalling pathway plays a critical role in regulating cell competition. YAP overexpression or promotion of YAP nuclear localization in cells can alleviate their ‘loser’ phenotype [19, 37–39]. We found that fibroblasts in the microenvironment of bone repair enhanced the cytoplasmic translocation of YAP in MSCs by activating the Hippo signalling pathway to induce MSCs to show the ‘loser type’. As a result, the proliferation of MSCs was inhibited. Additionally, MSCs could not further differentiate into osteoblasts, and preosteoblasts were eliminated, both leading to inactive osteogenesis in the fibrous scars.

Cell competition can occur via metabolic or structural cell competition [9, 20]. YAP/TAZ can sense changes in the competition of space and nutrients between cells [40]. It has been reported that YAP drives cell competition between variant human pluripotent stem cells (hPSCs) in mosaic cultures. When YAP is sequestered in the cytoplasm, wild-type cells undergo apoptosis, while recurrent culture-acquired aneuploidies obtain growth advantages [38]. YAP also drives cell competition in cancers. High expression levels of YAP have been shown to induce dominant clones when glioma cells are grown in two-dimensional culture conditions or as tumour spheroids [37]. In addition, YAP has been found to regulate cell competition via metabolism [41]. In this project, we found that fibroblasts competed with MSCs independently of direct contact. Fibroblasts induced the nuclear-cytoplasmic shuttling of YAP in MSCs whenever they were in direct or indirect contact. When fibroblasts and MSCs were in direct contact, they shared both space and nutrients. However, the inhibitory effects still existed when fibroblasts and MSCs were cocultured separately in Transwells. In this condition, only the exchange of culture medium through the permeable membrane of the Transwell was allowed. Thus, both limited space and nutrients might contribute to the competition between MSCs and fibroblasts.

YAP and TAZ act as coactivators for the transcription factor TEAD, sharing many functions. Studies have shown that both YAP and TAZ are crucial for inducing osteogenic differentiation of MSCs [11, 42, 43]. YAP and TAZ might play redundant roles in osteogenic differentiation. This notion is supported by Zhao et al.’s observation that YAP knockout blocked neural nest cell osteoblast differentiation, similar to YAP/TAZ double knockdown [44]. In this study, we found that fibroblasts could regulate the nuclear-cytoplasmic localization of YAP in MSCs, thereby hindering the ability of MSCs to differentiate into osteoblasts. Interestingly, knocking down YAP alone significantly impeded the osteoblast differentiation of MSCs even if TAZ expression was not disturbed. Conversely, nuclear localized YAP could fully restore osteoblast differentiation of MSCs despite the presence of fibroblasts. Previous reports showed that knockout of YAP in a mouse model resulted in bone loss and inhibition of osteoblast differentiation in vivo [11]. Moreover, another study showed that YAP knockdown inhibited the osteogenic differentiation of MSCs, while overexpression of YAP enhanced this process [45], which was consistent with our results. These findings and our results suggest that YAP may play a unique role in osteogenesis, since knocking down YAP abrogates MSC osteogenic differentiation, and TAZ is unable to rescue YAP knockdown. MSCs may require sufficient levels of YAP and TAZ for full osteogenic differentiation.

Fibroblasts can regulate osteogenesis via diverse mechanisms. As mentioned above, microRNAs and cytokines secreted by fibroblasts also contribute to the inhibition of osteogenesis [7, 8]. In this project, we discovered that fibroblasts secreted a natural Wnt signal inhibitor, DKK1, to further inhibit osteogenesis. Wnt/β-Catenin signalling has been proven to play important roles in bone development and osteogenesis [28, 46]. In recent years, targeting Wnt signalling to enhance osteogenesis has remained a hot spot in bone healing research [27, 35]. Anti-DKK1 has been reported to enhance osteogenic differentiation of MSCs [47], and miRNA-488, which targets DKK1, has also been proven to enhance bone healing [48]. We found that DKK1 could be a cytokine secreted by fibroblasts to inhibit osteogenesis. Targeting fibroblasts may provide another approach to inhibit DKK1 and activation of Wnt signalling, further enhancing osteogenesis.

The role of DKK1 in osteogenesis has been known for years. Bajada et al. identified a stromal cell population from bone nonunion tissues similar to MSCs, which could be differentiated into osteoblasts. However, compared with bone marrow-derived MSCs, the osteogenic differentiation capabilities of stromal cells from bone nonunion tissues were limited due to DKK1 autocrine signalling [49]. Other reports have also demonstrated that knocking out DKK1 can enhance osteogenesis in different animal models [26, 47, 50, 51]. In contrast to previous reports, our study showed that fibroblasts could secrete DKK1 to target MSCs. Thus, DKK1 may originate from both autocrine and paracrine sources to target MSCs, ultimately playing a vital role in inhibiting the osteoblast differentiation of MSCs.

## Conclusion

Our research has revealed that fibroblasts can inhibit osteoblast differentiation of MSCs under coculture conditions. We found that this process occurs through two mechanisms. First, fibroblasts can regulate the nuclear-cytoplasmic shuttling of YAP in MSCs, which is crucial for regulating the osteoblast differentiation of MSCs. Second, fibroblasts secrete DKK1, which inhibits the formation of calcium nodules at the late stage of osteogenesis. Our findings also suggest that the accumulation of fibrous tissues is associated with the cytoplasmic localization of YAP in preosteoblasts and reduced osteogenesis in vivo. Taken together, these results indicate that fibroblasts can competitively inhibit the osteogenesis of MSCs, potentially leading to bone fracture nonunion.

### Supplementary Information

**Additional file 1: Table S1.** Characteristics of the five donors with bone nonunion. **Table S2.** Primer sequences of Quantitative real-time PCR. **Figure S1.** The effects of conditional media from the fibroblasts on BMP2 induced osteoblast differentiation of MSCs. **Figure S2.** YAP knocked down affected proliferation and osteoblast differentiation of MSCs. **Figure S3.** The effects of conditional media from fibroblasts on the nuclear-cytoplasmic localization of YAP in MSCs. **Figure S4.** The correlations among fibrous tissues accumulation, osteogenesis and YAP nuclear-cytoplasmic shuttling in human bone nonunion. **Figure S5.** The correlations among fibrous tissues accumulation, osteogenesis and YAP nuclear-cytoplasmic shuttling in BMP2 induced ectopic osteogenesis. **Figure S6.** The correlations among fibrous tissues accumulation, osteogenesis and YAP nuclear-cytoplasmic shuttling in BMP2 induced ectopic osteogenesis. **Figure S7.** Original data of gels conducted by Western Blot.

## Data Availability

The dataset supporting the conclusions of this article is available at the Gene Expression Omnibus (GEO) (No. GSE205156).
