# Supplementary material for: Fibroblasts inhibit osteogenesis by regulating nuclear-cytoplasmic shuttling of YAP in mesenchymal stem cells and secreting DKK1
Source: Biol Res. 2024 Jan 20;57:4. doi: 10.1186/s40659-023-00481-y (PMC10799393; doi:10.1186/s40659-023-00481-y)
Supplement: Supplementary file 1 — Additional file 1: Table S1. Characteristics of the five donors with bone nonunion. Table S2. Primer sequences of Quantitative real-time PCR. Figure S1. The effects of conditional media from the fibroblasts on BMP2 induced osteoblast differentiation of MSCs. Figure S2. YAP knocked down affected proliferation and osteoblast differentiation of MSCs. Figure S3. The effects of conditional media from fibroblasts on the nuclear-cytoplasmic localization of YAP in MSCs. Figure S4. The correlations among fibrous tissues accumulation, osteogenesis and YAP nuclear-cytoplasmic shuttling in human bone nonunion. Figure S5. The correlations among fibrous tissues accumulation, osteogenesis and YAP nuclear-cytoplasmic shuttling in BMP2 induced ectopic osteogenesis. Figure S6. The correlations among fibrous tissues accumulation, osteogenesis and YAP nuclear-cytoplasmic shuttling in BMP2 induced ectopic osteogenesis. Figure S7. Original data of gels conducted by Western Blot. [file 40659_2023_481_MOESM1_ESM.docx]

**Additional file 1**

**Fibroblasts Inhibit Osteogenesis by Regulating Nuclear-Cytoplasmic Shuttling of YAP in Mesenchymal Stem Cells and Secreting DKK1**

Fei Huang^c^, Guozhen Wei^a,b^, Hai Wang^a,b^, Ying Zhang^c^, Wenbin Lan^a,b^, Yun Xie^a,b*^, Gui Wu^a,b*^

^a^Department of Orthopaedics, the First Affiliated Hospital, Fujian Medical University, Fuzhou, Fujian, 350005, China

^b^Department of Orthopaedics, National Regional Medical Center, Binhai Campus of the First Affiliated Hospital, Fujian Medical University, Fuzhou, Fujian, 350212, China

^c^Central Laboratory, First Affiliated Hospital, Fujian Medical University, Fuzhou, Fujian, 350005, China.

*Corresponding author:

Gui Wu, MD

Department of Orthopaedics,

the First Affiliated Hospital of Fujian Medical University,

No. 20, Chazhong Road, Taijiang District, Fuzhou, 350005

China ­

Email: wugui_1985@sina.com

Yun Xie, MD

Department of orthopaedics,

the First Affiliated Hospital of Fujian Medical University,

No. 20, Chazhong Road, Taijiang District, Fuzhou, 350005

China

Email: xyxlr@126.com

| Table S1. Characteristics of the five donors with bone nonunion | |
| --- | --- |
| Bone nonunion cases | n=5 |
| **Age**/yr |  |
| Average | 39.4 |
| Range | 27-57 |
| **Sex**/No. patients (number) |  |
| Female | 2 |
| Male | 3 |
| **Location of bone fracture nonunion**/ No. patients (number) |  |
| Femoral | 3 |
| Tibial | 2 |
| **Time from fractures to non-union**/ months |  |
| Average | 16 |
| Range | 7-36 |
| **Other concomitant diseases**/ No. patients (number) |  |
| Yes | 0 |
| No | 5 |

**Table S2 Primer sequences of Quantitative real-time PCR**

| Gene Names | Forward (5’-3’) | Reverse (5’-3’) |
| --- | --- | --- |
| *Runx2* | AGAGTCAGATTACAGATCCCAGG | TGGCTCTTCTTACTGAGAGAGG |
| *Ocn* | CCACCCGGGAGCAGTGT | CTAAATAGTGATACCGTAGATGCGTTTG |
| *Osx* | TCTCAAGCACCAATGGACTCCT | GGGTAGTCATTTGCATAGCCAGA |
| *Gapdh* | CATGGCCTTCCGTGTTCCTA | CCTGCTTCACCACCTTCTTGAT |

**S1**

**
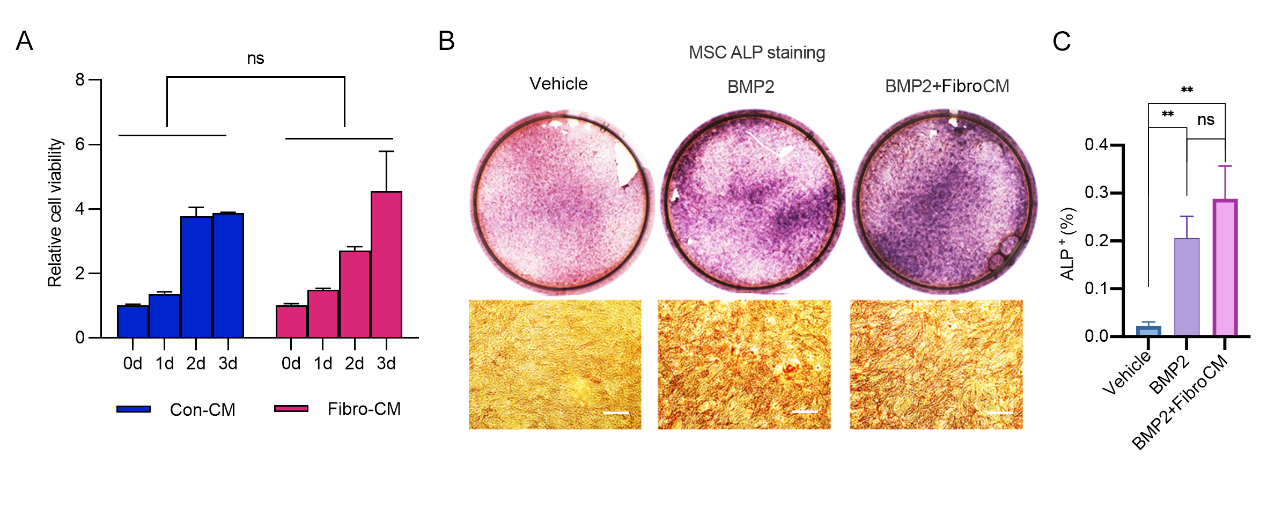
**

**Figure S1. The effects of conditional media from the fibroblasts on BMP2 induced osteoblast differentiation of MSCs.**

1. A total of 3×10^3^ MSCs were treated with conditional media from fibroblasts for the indicated time point. Subsequently, the cells were harvested and subjected to the CCK-8 assay. The experiments were conducted for three times, and the average relative cell viability to Day 0 was calculated and presented.
2. After 1×10^4^ MSCs were treated with conditional media from fibroblasts and BMP2 was utilized to induce osteogenic differentiation of MSCs for seven days, then cells were harvested and subjected to ALP staining. Representative images of wells and cells were presented. Scale bars, 100 μm.
3. The percentages of ALP^+^ cells in (B) were quantitatively analyzed. The average percentages of ALP+ cells were calculated from at least three fields and shown (***P <0.01*).

**S2**


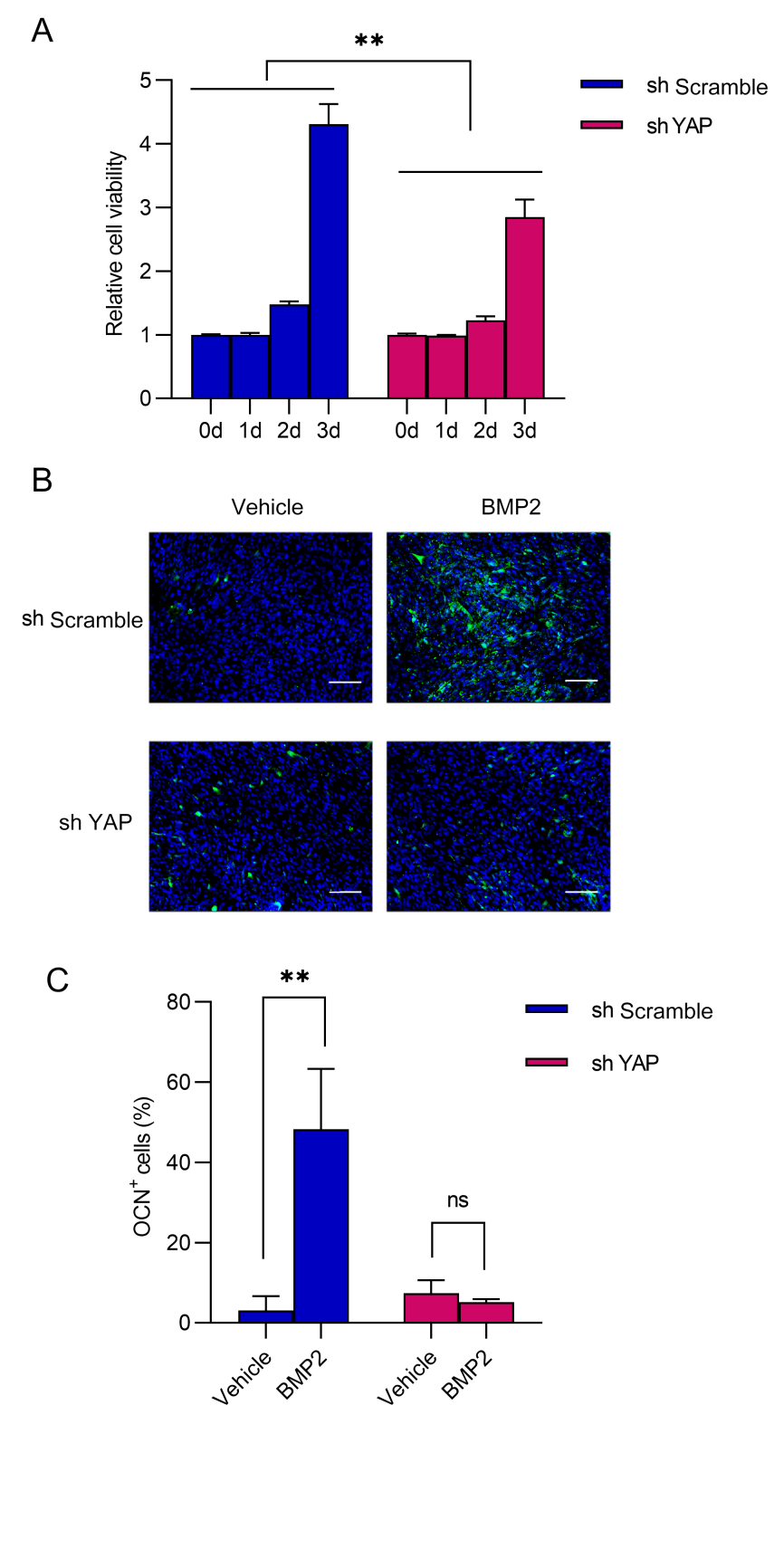


**Figure S2. YAP knocked down affected proliferation and osteoblast differentiation of MSCs.**

1. After YAP was knocked down, 3×10^3^ MSCs were cultured in a single well of a 96-well plate for the indicated time point. Subsequently, the cells were harvested and subjected to the CCK-8 assay. The experiments were conducted for three times and the average relative cell viability to Day 0 was calculated and presented (***P <0.01*).
2. After YAP was knocked down, 1×10^4^ MSCs were cultured either with a vehicle or with 200 ng/mL BMP2 for seven days. Subsequently, cells were harvested and subjected to immunofluorescent staining. OCN was marked green color, while DAPI was marked blue color. Representative images were presented. Scale bars, 100 μm.
3. The percentages of OCN^+^ cells in (B) were quantitatively analyzed. The average percentages of OCN^+^ cells were calculated from at least three fields and shown (**P<0.05; **P <0.01*).

**S3**

**
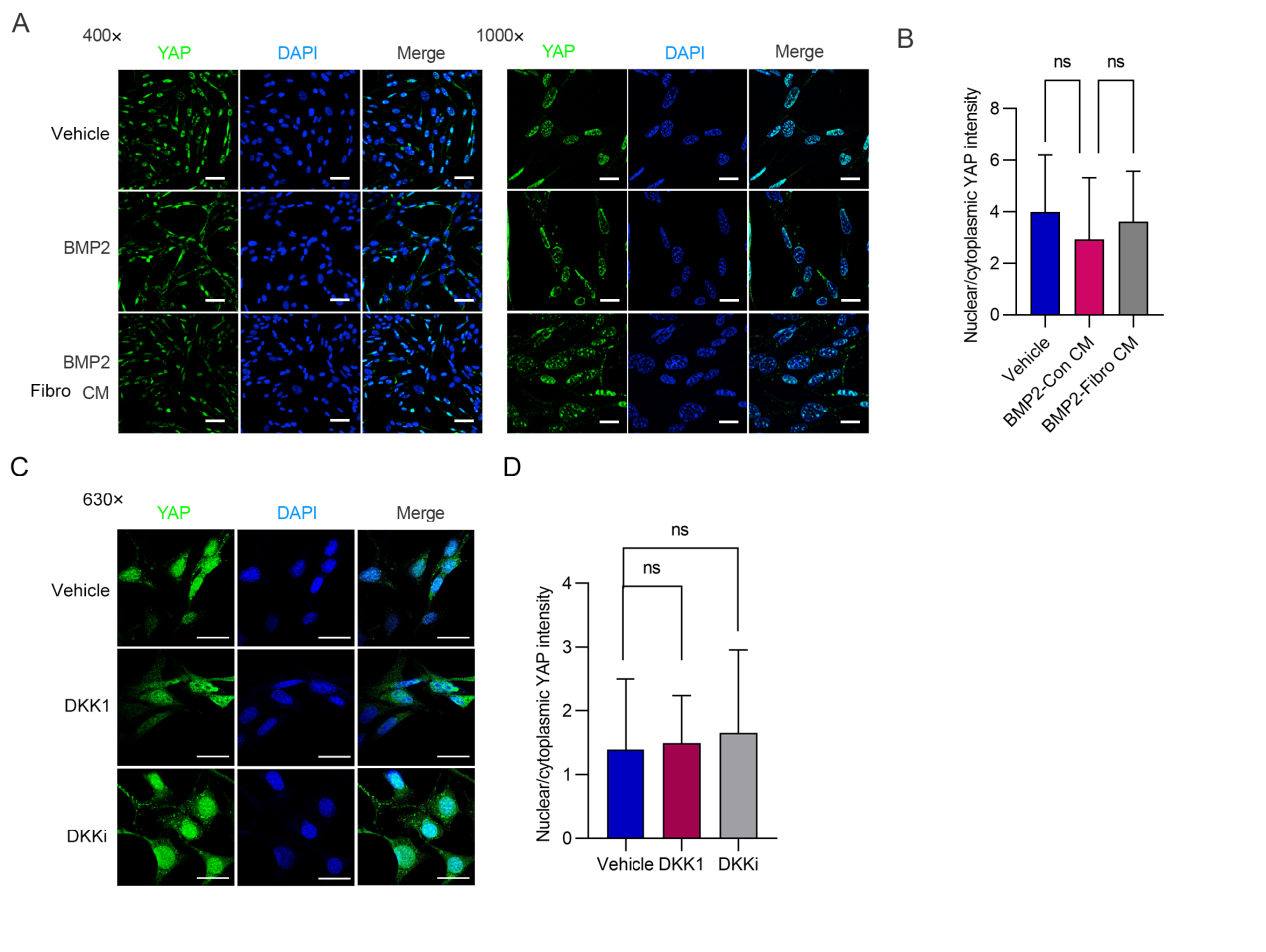
**

**Figure S3. The effects of conditional media from fibroblasts on the nuclear-cytoplasmic localization of YAP in MSCs.**

A. After being treated with a vehicle, 200 ng/mL BMP2 or conditional media from fibroblasts for two hours, MSCs were harvested and subjected to immunofluorescent staining. Representative immunofluorescent staining images of MSCs were shown. Green color represented YAP, while blue color indicated DAPI. Scale bars, 25 μm for 400×, 10μm for 1000×.

B. Quantitative analysis of nuclear/cytoplasmic YAP intensity of MSCs in (A) was shown. The average nuclear/cytoplasmic YAP intensity of at least ten cells were calculated and presented.

C. After being treated with a vehicle, 10 ng/mL DKK1 or 5 μM gallocyanine (DKKi) for two hours, MSCs were harvested and subjected to immunofluorescent staining. Representative immunofluorescent staining images of MSCs were shown. Green color represented YAP, while blue color indicated DAPI. Scale bars, 10 μm.

D. Quantitative analysis of nuclear/cytoplasmic YAP intensity of MSCs in (C) was shown. The average nuclear/cytoplasmic YAP intensity of at least ten cells were calculated and presented.

**S4**

**
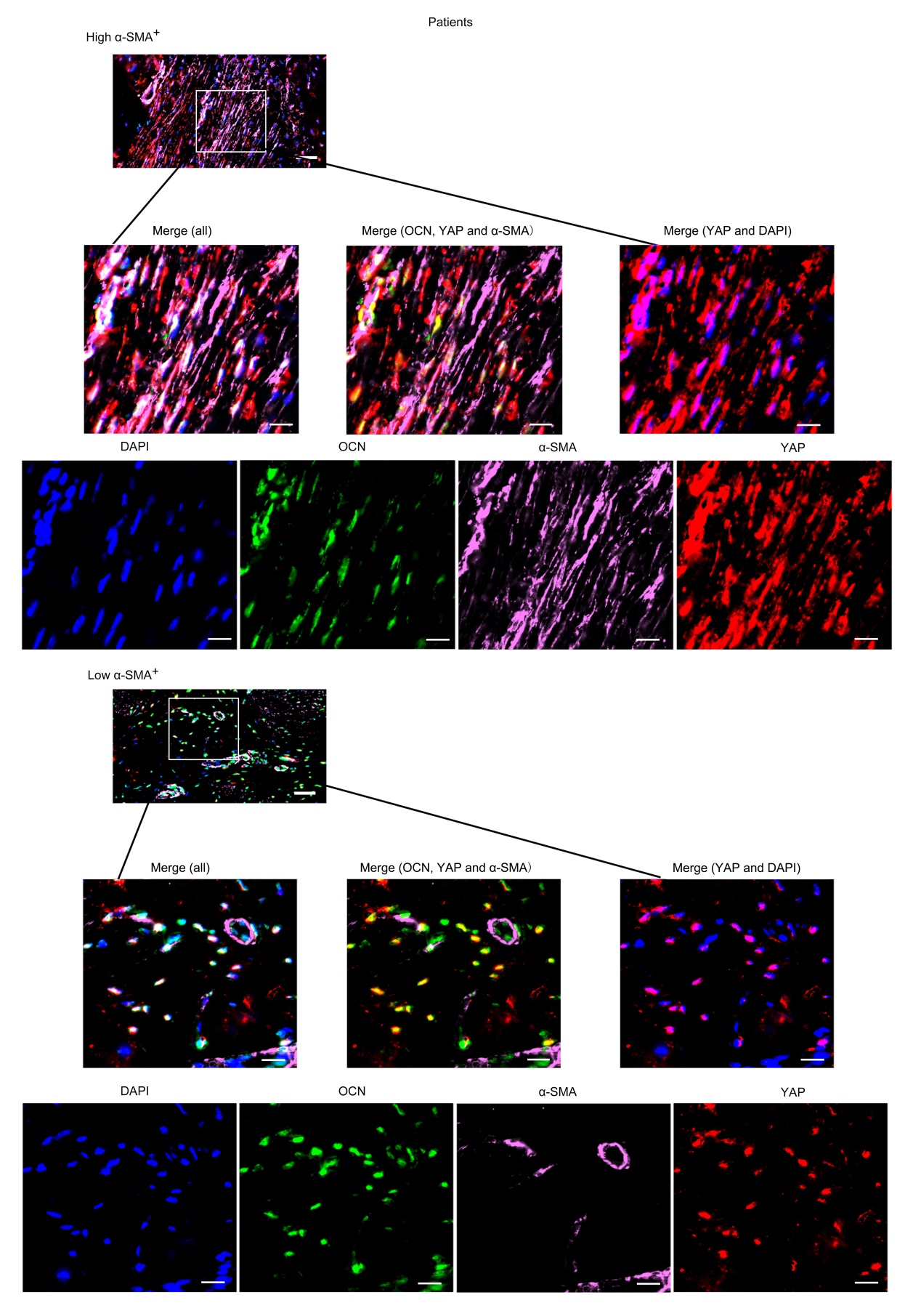
**

**Figure S4. The correlations among fibrous tissues accumulation, osteogenesis and YAP nuclear-cytoplasmic shuttling in human bone nonunion**

Tissues obtained from patients with nonunion of fractures were harvested and sliced into multiple sections. These sections were subsequently divided into several small unites to enable thorough analysis following immunofluorescent staining. If the α-SMA positive area was over 50%, the area was classified as the high α-SMA expression area, while less than 50% was considered as low expression. Magnification of immunofluorescent staining images in Fig. 6A were shown. DAPI, OCN and YAP were also stained as indicated in the images. Scale bars, 20 μm.

**S5**

**
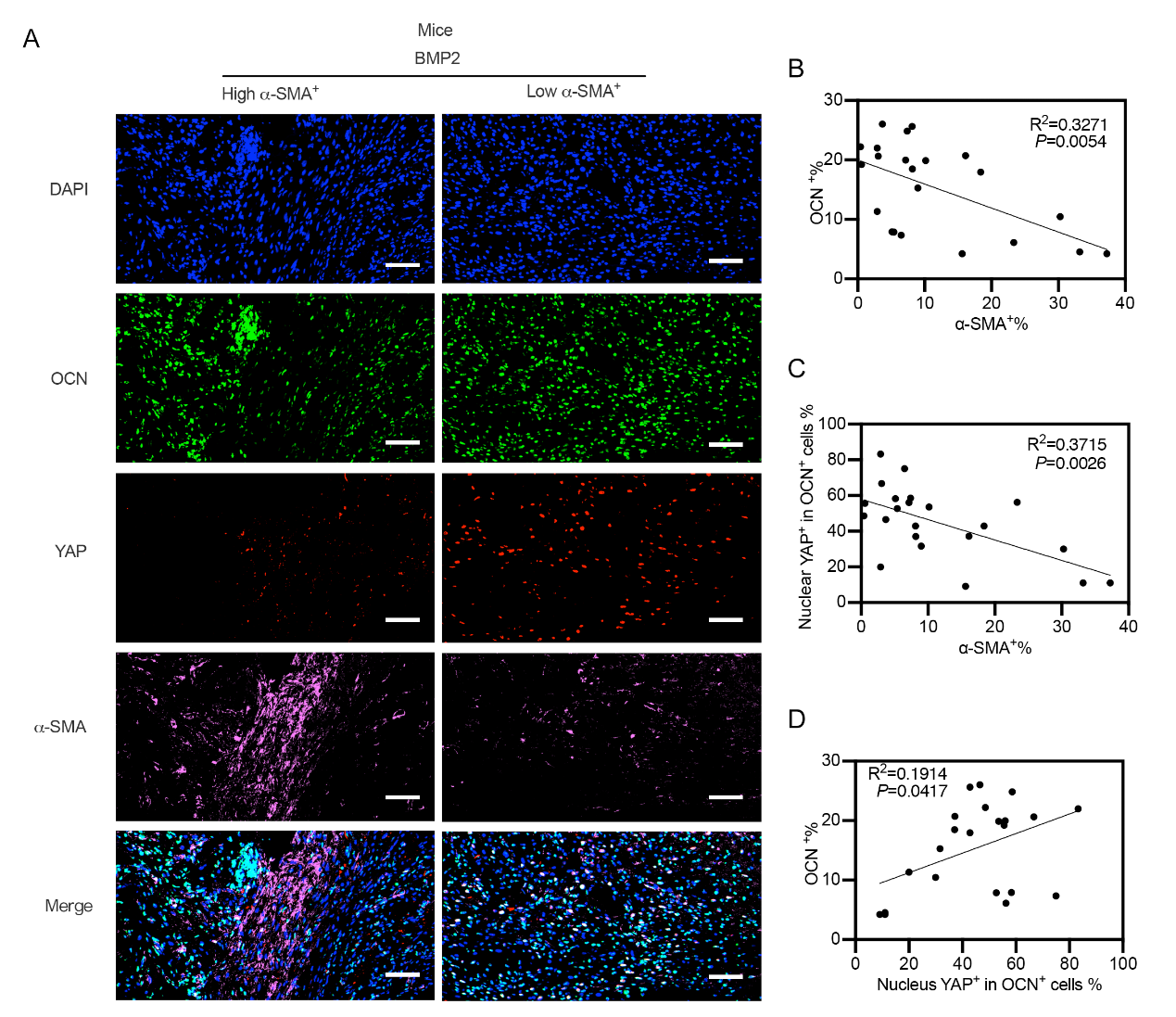
**

**Figure S5. The correlations among fibrous tissues accumulation, osteogenesis and YAP nuclear-cytoplasmic shuttling in BMP2 induced ectopic osteogenesis.**

A. BMP2/collagen gels were implanted into the muscles of mice for a period of 8 weeks. The resulting tissues were harvested for analysis. Each section of immunofluorescent staining image was segmented into multiple small unites for further analysis. If the α-SMA positive area was over 50%, the area was classified as the high α-SMA expression area, while less than 50% was considered as low expression. Representative immunofluorescent staining images either with high or with low α-SMA^+^ expression were shown. DAPI, OCN and YAP were also stained as indicated in the images. Scale bars, 100 μm.

B. Scatter diagram illustrated the correlation between the percentage of α-SMA^+^ areas and the percentage of OCN^+^ areas in (A). We ensured accuracy by having at least three different experimenters analyze all images, with the average value of each experiment being shown.

C. The nuclear and cytoplasmic YAP intensity in OCN positive cells were calculated using the software Image J. Nuclear YAP positive cells were defined as cells in which the nuclear YAP intensity/cytoplasmic YAP intensity was over 1. Scatter diagram illustrated the correlation between the percentage of α-SMA^+^ areas and the percentage of nuclear YAP^+^ in OCN^+^ cells in (A). We ensured accuracy by having at least three different experimenters analyze all images, with the average value of each experiment being shown.

D. Scatter diagram illustrated the correlation between the percentage of OCN^+^ areas and the percentage of nuclear YAP^+^ in OCN^+^ cells in (A). We ensured accuracy by having at least three different experimenters analyze all images, with the average value of each experiment being shown.

**S6**


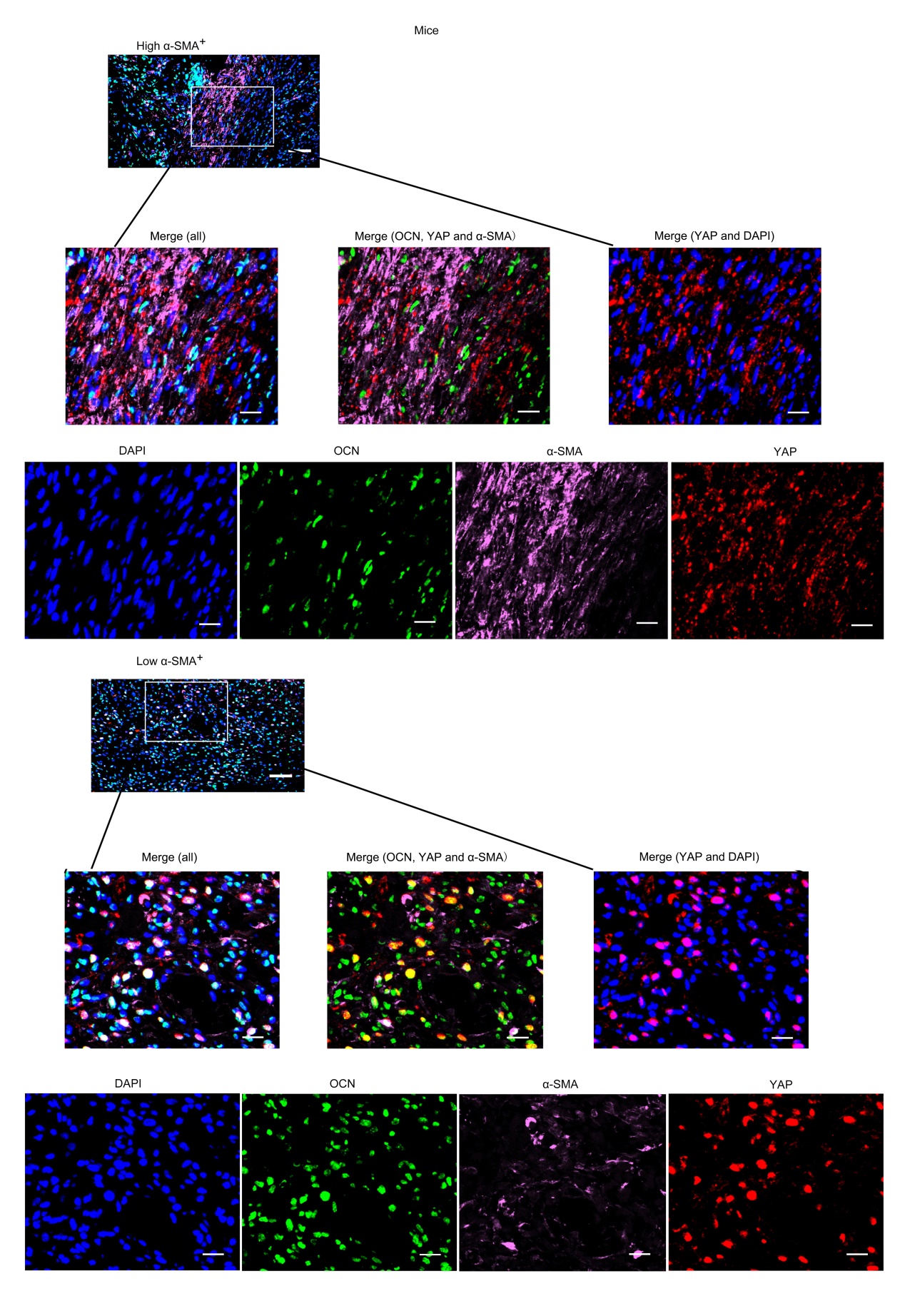


**Figure S6. The correlations among fibrous tissues accumulation, osteogenesis and YAP nuclear-cytoplasmic shuttling in BMP2 induced ectopic osteogenesis.**

BMP2/collagen gels were implanted into the muscles of mice for a period of 8 weeks. The resulting tissues were harvested for analysis. Each section of immunofluorescent staining image was segmented into multiple small unites for further analysis. If the α-SMA positive area was over 50%, the area was classified as the high α-SMA expression area, while less than 50% was considered as low expression. Magnification of immunofluorescent staining images in Fig. S5A were shown. DAPI, OCN and YAP were also stained as indicated in the images. Scale bars, 20 μm.

**S7**


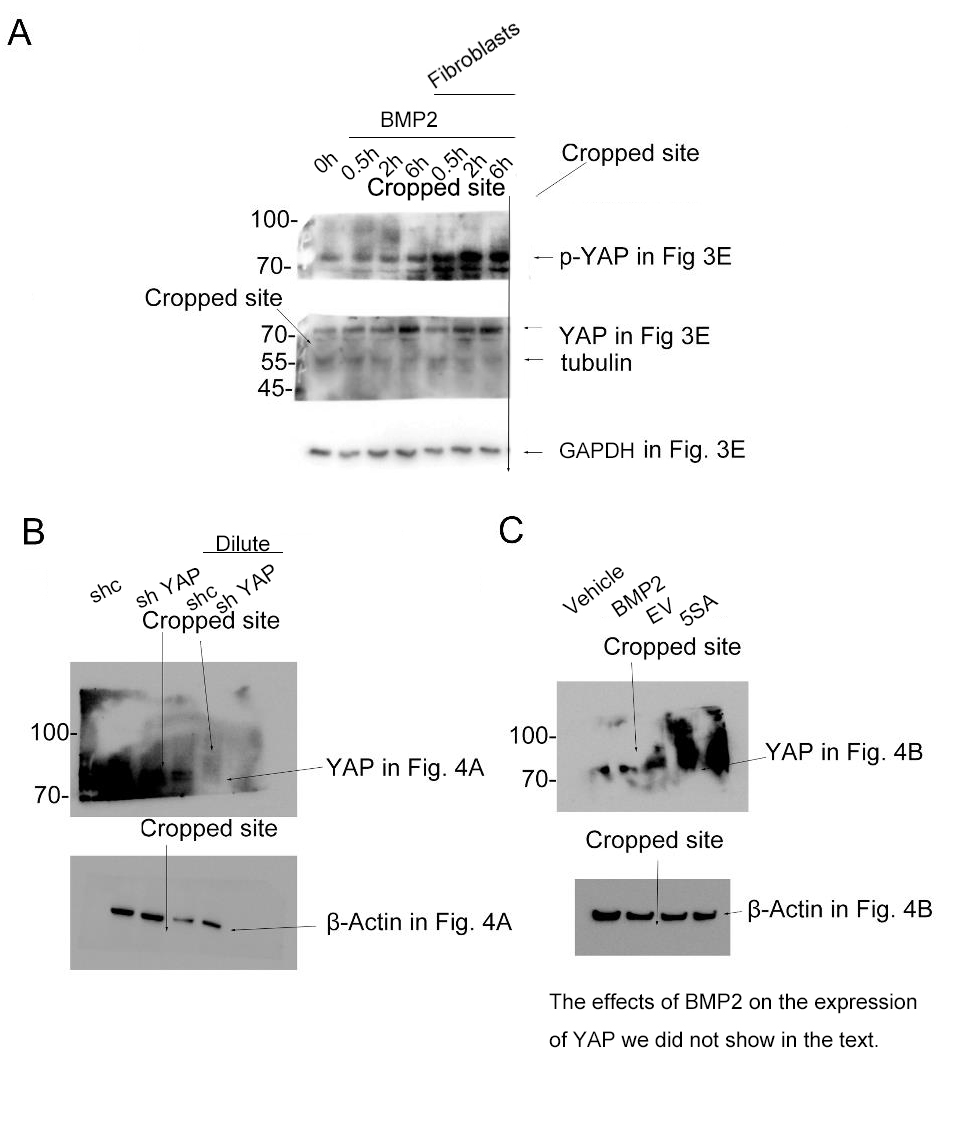


**Figure S7**. Original data of gels conducted by Western Blot.

1. Original data of gels in Fig. 3E.
2. Original data of gels in Fig. 4A.
3. Original data of gels in Fig. 4B.
